# Oxide Growth and Place-Exchange on Au(111) in Alkaline Electrolyte

**DOI:** 10.1021/acselectrochem.5c00505

**Published:** 2026-02-03

**Authors:** Toni Moser, Francesc Valls Mascaró, Andrea Auer, Julia Kunze-Liebhäuser

**Affiliations:** 27255 University of Innsbruck, Innrain 52c, 6020 Innsbruck, Austria

**Keywords:** gold, Au(111), electrochemical scanning tunneling microscopy (EC-STM), noble metal, oxidation, place-exchange, dissolution, oxide growth front, adatom and vacancy islands, surface roughening

## Abstract

Understanding the oxidation processes of noble metals is essential for the elaborate design of functional and stable materials for electrochemical applications, where electrocatalysis is currently central due to the need for efficient direct energy conversion devices. Single crystalline gold (Au(111)) is an important noble metal standard, as it has high relevance as an electrocatalyst material and is best suited for the study of fundamental surface and interface processes due to its high nobility and the model applicability of the processes at its electrified solid/liquid interface. Under electrochemical conditions, Au(111) oxidation proceeds via a place-exchange mechanism, in which surface Au atoms exchange positions with adsorbed oxygen species. While this process is well studied in acidic media, where it results in the nucleation and growth of adatom and vacancy islands alongside partial dissolution, its behavior in alkaline media remains less explored. Here, we investigate the oxidation of Au(111) in 0.1 M NaOH, providing new insights into the oxidation process and associated surface restructuring mechanisms. Using electrochemical scanning tunneling microscopy, we directly observe the growth of a surface oxide layer across the Au(111) terraces, which reveals the slow, kinetically limited dynamics of the place-exchange process. Once this place-exchange process has occurred, the pristine surface structure is not restored upon electrochemical reduction within experimental time scales, as vacancy islands form and persist. These findings are crucial for developing strategies to mitigate catalyst degradation and enhance the stability of Au-based materials in electrochemical applications.

## Graphical abstract

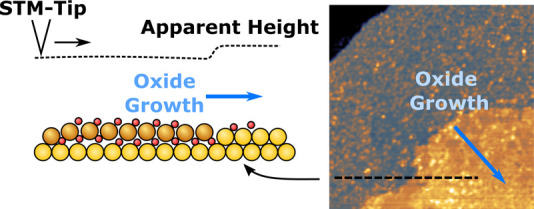

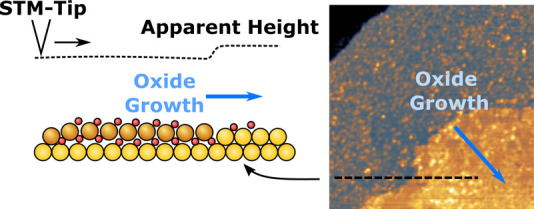

## Introduction

The electrochemical oxidation of noble metals has been investigated for over 50 years due to its profound impact on the nature of the solid/liquid interface,^1–4^ which majorly influences the properties of electrode materials and thus their performance in significant applications such as electrocatalysis. Understanding the surface oxide formation mechanism is therefore crucial not only for controlling electrocatalytic activity and selectivity but also for ensuring long-term catalyst stability. Profound knowledge of oxidation processes would consequently facilitate both design and operational control of (composite) catalyst materials and ensure their enhanced and durable performance.

Several authors proposed that the surface oxidation process begins with the so-called place-exchange mechanism, in which surface atoms interchange their position with previously adsorbed oxygen atoms (O_ads_) that diffuse subsurface.^4–8^ This mechanism has been extensively investigated on platinum (Pt), where it is well-stablished that it occurs in two distinct steps: reversible and irreversible place-exchange.^9,10^ At low oxidation potentials, Pt surface atoms are vertically displaced upwards by nearly one atomic step height while O_ads_ atoms move subsurface.^11–13^ At this early stage, a subsequent reduction of the surface does not lead to structural changes, as all atoms go back to their original positions; this step is named *reversible place-exchange*.^11,13–15^ In contrast, at higher i.e., more positive oxidation potentials, the surface stress, caused by the larger lattice parameters of the evolving surface oxide compared to the metallic surface, becomes significant enough that Pt surface atoms are expelled onto the terrace as adatoms.^11,13,16^ Upon reduction, this process induces irreversible surface structure changes, disrupting its original integrity through the formation of adatom and vacancy islands.^13,17^ The irreversibility stems from kinetic constraints, specifically the limited interlayer diffusivity of adatoms at typical experimental conditions, which prevents the surface from recovering its initial structural configuration. This kinetically hindered restoration is the basis for the term *irreversible place-exchange*.

The electrochemical oxidation of gold (Au) is much less studied, despite its critical role in key catalytic reactions such as the oxidation of carbon monoxide^18–20^ and larger organic molecules like alcohols^21^ and alkenes.^22^ Surface X-ray diffraction measurements on Au(111) suggest that the place-exchange occurs.^23–25^ However, the surface oxide structure could not be resolved, which limits the data interpretation and leads to the conclusion that the place-exchange likely proceeds in a disordered manner.^25^ A detailed understanding of this process does therefore not exist at the moment. In addition, the distinction between reversible and irreversible place-exchange has never been clearly addressed on Au. Early electrochemical scanning tunneling microscopy (EC-STM) studies indicate that in 0.1 M H_2_SO_4_ the pristine Au(111) surface is restored if the potential is reversed at 1.46 V_RHE_, while adatom and vacancy islands form if the oxidation potential reaches 1.6 V_RHE_.^26^ Similar studies in 0.1 M HClO_4_ revealed that pits appear only when the potential exceeds 1.44 V_RHE_ prior to reducing the surface oxide.^27^ However, a distinct link between surface degradation, oxidation state, and structure is still missing.

The numerous EC-STM studies on the oxidation and reduction of Au(111)^26–35^ have almost exclusively been conducted in acidic media. The sole investigation of Au(111) in alkaline media^35^ focused on surface healing after oxidation and reduction, while the oxidation process itself and its potential-dependent structural details remained unexplored. This lack of systematic investigation of the Au oxidation in alkaline media is surprising, especially because CO oxidation is strongly promoted at high pH values.^20^

The anodic voltammetric fingerprint of Au(111) in alkaline electrolyte differs significantly from that observed in acidic environments, which indicates that its oxidation proceeds via different mechanistic pathways. In alkaline electrolytes, OH adsorption occurs at moderately positive potentials and induces the lifting of the Herringbone (HB) reconstruction.^36–38^ Under acidic conditions, OH adsorption is thermodynamically shifted to higher potentials due to the lower pH. In electrolytes containing specifically adsorbing anions (e.g., sulfate), competitive anion adsorption causes a further increase of this overpotential.^4^ Beyond these thermodynamically controlled adsorption characteristics, alkaline conditions also enhance surface mobility, which results in less rough surfaces after oxidation and reduction.^35^ Moreover, Au dissolution is substantially enhanced in alkaline media below the onset of the oxygen evolution reaction (OER), as revealed by inductively coupled plasma mass spectrometry (ICP-MS) studies.^39^ Similar to the case of Pt, where both anodic and cathodic dissolution were shown to be intrinsic to the place-exchange mechanism,^40^ the surface roughening of Au^41^ is also intimately linked to this phenomenon. Therefore, a clear elucidation of the oxidation behavior of Au in alkaline electrolytes is critical for a comprehensive understanding of these processes.

In this article, we use EC-STM to investigate the details of the electrochemical oxidation process of the Au(111) surface with the related surface structural changes in 0.1 M NaOH. OH adsorbates appear as precursors of oxide formation in patches of lower apparent height on the surface, while the subsequent conversion of OH_ads_ to O_ads_—correlated with a distinct anodic peak that clearly corresponds to a charge transfer process—is accompanied by a pronounced increase in surface roughness. At sufficiently positive potentials after the O_ads_ formation peak, we observe the growth of a surface oxide in form of a propagating growth front that appears to move across the entire surface imaged with EC-STM. This process results in the ejection of surface atoms and in a measurable decrease of the surface roughness. After this surface restructuring, the pristine surface is not fully restored upon reduction, as vacancy islands form and remain metastable even at high negative potentials. Our findings thus provide detailed structural insight into the oxidation process of Au(111), revealing the growth of an oxide layer that is closely associated with irreversible phenomena such as dissolution and place exchange.

## Experimental Section

### Au­(111) Preparation

The Au(111) single crystal (MaTecK, Ø = 10 mm, 99.999% purity, polished to <0.1°) was first cleaned using freshly prepared concentrated Caro’s acid, followed by repeated rinsing and boiling, at least three times, in ultrapure water (Milli-Q, Merck, 18.2 MΩ·cm). The crystal was then flame-annealed using a propane flame until it reached an orange glow (∼3 min) and subsequently cooled under a continuous flow of high-purity argon (Ar) gas (Messer, 99.999%).

### EC-STM Studies

STM tips were fabricated by electrochemical etching of a Pt_80_Ir_20_ wire (Goodfellow, Ø = 0.25 mm) in a 4 M KSCN and 2 M KOH solution. After etching, the tips were insulated with Apiezon wax. EC-STM measurements were conducted using a Bruker Multimode 8 scanning tunneling microscope housed within an Ar-filled glovebox (MBraun MB 200 MOD), where oxygen levels were consistently maintained below 5 ppm. Electrolyte solutions of 0.1 M NaOH (Merck, 99.99% trace metal basis) were degassed by purging with high-purity Ar to eliminate dissolved oxygen.

The Au(111) single crystal was mounted in a custom-designed EC-STM cell made of polychlorotrifluoroethylene (PCTFE), which had been cleaned using Caro’s acid and subsequently rinsed and boiled multiple times in ultrapure water. Polytetrafluoroethylene (PTFE)-bound activated carbon served as both the quasi-reference and counter electrodes, following established procedures.^42^ All potentials are reported versus the reversible hydrogen electrode (RHE) for consistency with literature values.

Prior to each measurement, the cleanliness and structural integrity of the Au(111) surface were verified by imaging the characteristic √3 × 22 HB reconstruction via STM. Image analysis and visualization were performed using Gwyddion software.^43^

## Results and Discussion

### Cyclic Voltammetry of Au(111)

To establish a reference framework for the surface processes discussed below, we first analyze the cyclic voltammetric behavior of Au(111) in both acidic and alkaline media. Although the focus of this study is on the oxidation and reduction of Au(111) in 0.1 M NaOH, the comparison of the cyclic voltammograms (CVs) recorded in acidic and alkaline electrolytes provides valuable context, since the electrochemical response of Au(111) strongly depends on the pH, which influences both the potential range and mechanism of oxide formation and reduction. Figure 1 shows the CVs of Au(111) recorded in 0.1 M H_2_SO_4_ (panel A) and 0.1 M NaOH (panel B) inside the EC-STM cell. The low currents observed below 0.5 V_RHE_ are attributed to the capacitive charging/discharging of the double layer at the HB reconstructed surface.^36–38,44^ Above this potential, the differences between acidic and alkaline electrolytes are clearly visible.

**Figure 1:**
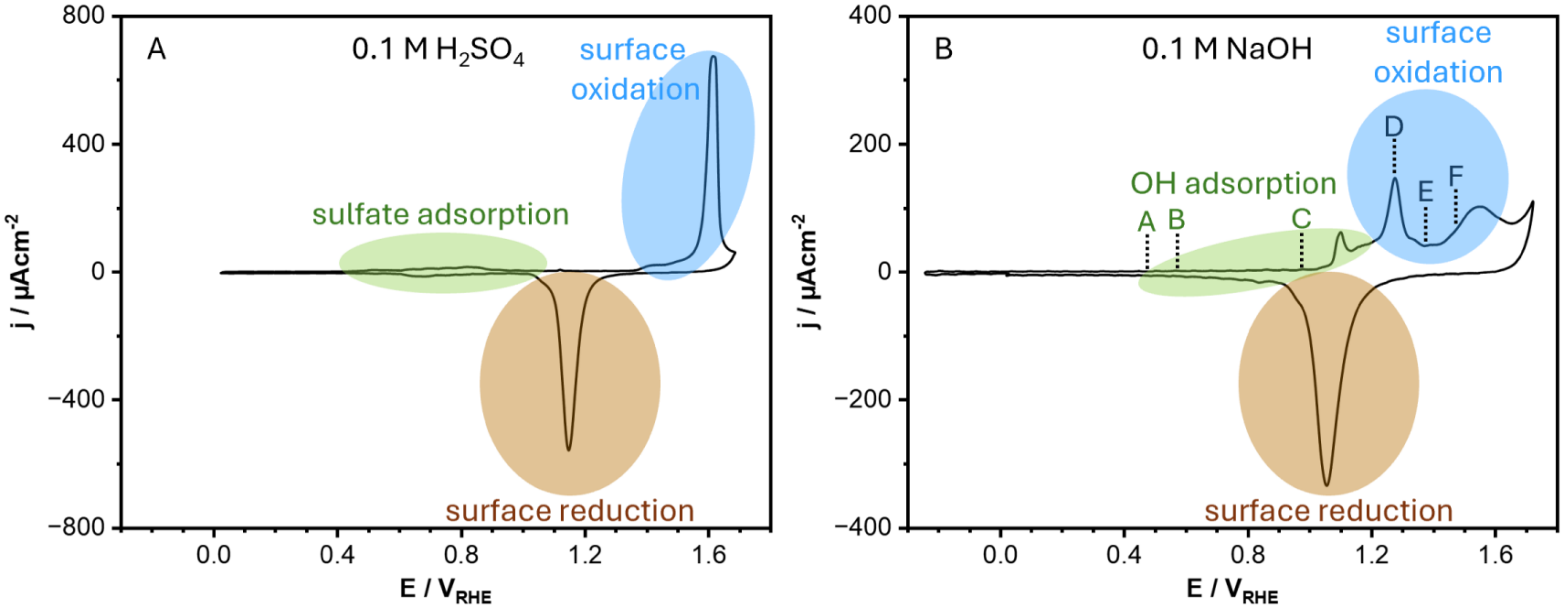
Cyclic voltammograms (CVs) of Au(111) in acidic (A) and alkaline (B) media. The colored labels (A–F) in panel (B) indicate the potentials at which we recorded the EC-STM images shown in Figure 2. The scan rate is 50 mV/s. In Figure S1, in the Supporting Information, we provide the full-scale CVs together with the corresponding apparent oxidation charges.

In 0.1 M H_2_SO_4_, adsorbed sulfate anions form an ordered adlayer on the Au(111) surface that competitively inhibits the adsorption of oxygen-containing species, thereby delaying the onset of surface oxidation.^4^ The anodic region of the CV exhibits two distinct peaks: at 1.4 V_RHE_ and at 1.6 V_RHE_. The first one is attributed to the oxidation of step (defect) sites.^45^ The second peak corresponds to the oxidation of the Au(111) terraces to form AuOOH,^45–47^ which is highly unstable and decomposes into Au_2_O_3_ ^47–50^ . This process is coupled with the so-called place-exchange mechanism, wherein Au terrace atoms are lifted and O_ads_ atoms move subsurface.^1,4–8,23–25,51–53^

In 0.1 M NaOH, the oxidation of the surface is preceded by OH adsorption, which causes a deviation of the current from the capacitive baseline and the anodic peak at 1.10 V_RHE_. In addition, it triggers the lifting of the HB reconstruction.^38,54,55^

The true oxidation of the Au(111) terraces begins with the anodic peak located at 1.27 V_RHE_, which corresponds to the dehydrogenation of OH_ads_ into O_ads_ according to

1 $$OH_{\mathrm{ads}}+OH^{-}\to O_{\mathrm{ads}}+H_{2}O+e^{-}$$

This was evidenced by infrared (IR) and Raman spectroscopy measurements.^45,46,54–56^ SXRD studies indicate that in alkaline electrolytes, similarly to what was observed in acidic media, O_ads_ diffuses subsurface.^55^ However, the precise details of this process, such as whether it occurs immediately after dehydrogenation or requires time to take place, remain unclear.

The third anodic peak, observed at around 1.5 V_RHE_, has been much less investigated in alkaline media. It is likely associated with the further oxidation of the terraces and/or the increase of the oxide layer thickness.^48^ Previous in-situ electrochemical XPS investigations from our group, however, indicate that, on polycrystalline Au in 0.1 M NaOH, Au^+^ is the predominant surface species at 1.57 V_RHE_, while Au^3+^ only appears at more positive potentials during the OER.^57^

In the cathodic scan, the reduction of the surface oxide occurs mainly through a single cathodic peak that is centered at 1.15 V_RHE_ in 0.1 M H_2_SO_4_, and at 1.05 V_RHE_ in 0.1 M NaOH.

### Electrochemical Oxidation of the Au(111) Surface

To understand the structural changes related to the anodic features that range from OH adsorption to oxidation at moderate overpotentials, we performed EC-STM investigations at the potentials labeled as A–F in Figure 1B. Figure 2 shows the respective EC-STM images of the Au(111) surface recorded in 0.1 M NaOH. The presence of the HB reconstruction at 0.47 V_RHE_ (see Figure 2A and its inset image for higher contrast) confirms the cleanliness of both the surface and the electrolyte.^37,38,44,58^ In the top-left corner of the image, two atomic steps are visible, with an apparent height of approximately 0.24 nm (see the height profile in Figure S2 in the Supporting Information). This value is in excellent agreement with the theoretical step height of 0.236 nm.

**Figure 2:**
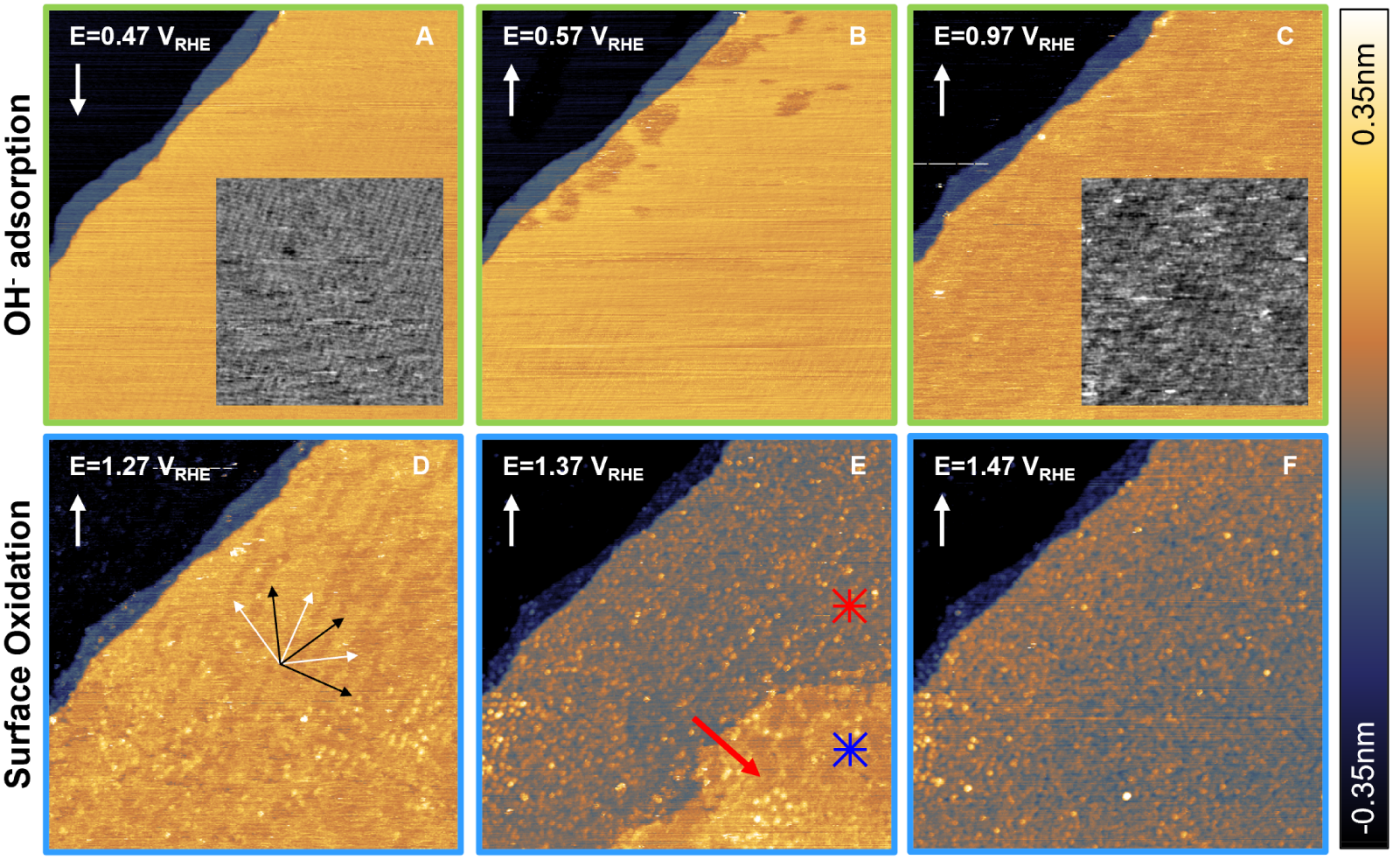
Structural evolution of the Au(111) surface during oxidation. EC-STM images recorded in 0.1 M NaOH upon anodic polarization in the OH adsorption regime (green frames) at (A) 0.47 V_RHE_, (B) 0.57 V_RHE_, and (C) 0.97 V_RHE_, as well as in the oxide formation regime (blue frames) at (D) 1.27 V_RHE_, (E) 1.37 V_RHE_, and (F) 1.47 V_RHE_. The grayscale insets in (A) and (C) are high-contrast overlays of the same positions. In (D), the black and white arrows indicate the main in-plane directions of the (111) surface and the ridge directions of the HB reconstruction, respectively. The red arrow in (E) highlights the direction of propagation of the oxide growth front. The asterisks indicate the area before (blue) and after (red) oxidation. Figures S3 and S4, in the Supporting Information, show additional images at intermediate potentials and the reduction series, respectively. White arrows in the top left corner of each image indicate the slow scan direction. All images are 200 × 200 nm^2^, recorded with *I*_tip_ = 1.8 nA and *E*_tip_ = 0.47 V_RHE_.

An increase of the potential to 0.57 V_RHE_ results in the adsorption of OH at the step edge, which appears as darker patches with an apparent depth of approximately 0.08 nm (see Figure 2B and Figure S2). This apparent height difference arises because Au covered with OH has a lower local density of states (LDOS) compared to the bare metal, causing it to appear lower. Similar observations have been reported for OH adsorption on Cu(111),^59^ for electrochemical oxidation of Au(111),^30^ and for O chemisorption on Al(111).^59,60^ Interestingly, in the proximity of these darker patches, the HB reconstruction appears to be lifted, highlighting the critical role of OH in lifting the HB reconstruction.^38^ However, unlike earlier observations with EC-STM in acidic media containing specifically adsorbing ions, Figure 2B does not show the formation of adatom islands from the 4–5% excess Au atoms expelled through the lifting of the reconstruction.^37,44,58^ This is likely because the reconstruction is lifted only locally, producing only a small number of adatoms that have sufficient time either to dissolve into the electrolyte or to incorporate into nearby step edges, which prevents the formation of stable islands. This effect is reinforced by the enhanced diffusion and dissolution kinetics in alkaline media.^35,39^

At 0.97 V_RHE_ the darker patches associated with OH_ads_ disappear, as shown in Figure 2C. The coverage of the surface with OH_ads_ amounts to less than 10% according to the approximate oxidation charge observed in Figure S1. This suggests that OH_ads_ is present and well distributed on the surface, even though it is hardly visible with STM. The literature on OH adsorption on Au(111) provides reasonable explanations for these findings. A detailed study of the Au(111) electrochemistry in alkaline media reported that OH_ads_ binds more polarly to the surface at low coverages, but the bond becomes more covalent as the potential increases.^54^ Consistent with this, density functional theory (DFT) calculations support that OH preferably adsorbs at three-fold hollow sites and transfers charge to the metal at positive potentials.^61^ This covalent bond weakens the repulsive interactions between OH_ads_ molecules, making them more mobile and effectively “invisible” to STM imaging.^62^ Interestingly, the HB reconstruction remains present at the surface at 0.97 V_RHE_ (Figure 2C), although it appears more disordered. This is likely due to the low local coverage with OH_ads_.

Figure 2D shows the Au(111) surface at the potential corresponding to the first oxidation peak maximum at 1.27 V_RHE_. At this potential, the HB reconstruction is lifted; however, dark lines remain along the [112̅] ridge direction of the HB reconstruction, as indicated by the white arrows, albeit with a significantly greater spacing than that of the typical HB pattern. This observation is consistent with the findings of Vaz-Domínguez et al. upon sulfate adsorption on Au(111).^44^ In the lower part of the image, oxide clusters are observed, likely originating from the excess surface atoms that are displaced onto the terraces during the complete lifting of the HB reconstruction at substantial parts of the surface. In Figure S5, in the Supporting Information, we show that a much larger density of well-distributed adatom islands forms upon rapidly stepping the potential from 0.49 V_RHE_ to 1.19 V_RHE_, which causes the immediate lifting of the HB reconstruction and leads to an increased adatom density due to too little time for the adatoms to either diffuse to the steps or dissolve.

More drastic changes occur at 1.37 V_RHE_ (see Figure 2E). The main terrace now consists of two distinct layers: a brighter region in the bottom-right corner of the image and a darker region closer to the step edge. The apparent height difference between these layers is approximately 0.12 nm, with the brighter region appearing higher (see Figure S6 in the Supporting Information). This contrast change is due to a decrease of the LDOS upon oxidation of the surface, rather than actual topographical differences. The darker appearing area spreads across the surface when the potential is increased beyond the first oxidation peak at 1.27 V_RHE_. This observation agrees with the findings of Vitus et al., who showed that, in acidic media at 1.55 V_RHE_, darker regions with an apparent height 0.12 nm lower than the unaffected area appear near step edges and then gradually spread across the Au(111) terraces.^30^ The growth mechanism of the surface oxide and the propagation of the growth front observed in Figure 2E as well as the interpretation of the related processes will be addressed in detail in a later section of this article.

At 1.47 V_RHE_ (Figure 2F), the growth front is no longer visible, as the whole terrace is now covered by a regular surface oxide. This assumption is supported by the fact that the oxidation charge at this potential, shown in Figure S1 in the Supporting Information, corresponds to 2.21 electrons per surface atom, which suggests the presence of one O per Au atom.

The morphological changes observed in Figure 2, i.e., from flat terraces to terraces covered by rough oxide, suggest a systematic evolution of the surface roughness with potential. To quantify this, we calculated the root mean square (RMS) roughness of the main terrace from each of the STM images measured. Details on this quantification are given in the Supporting Note 1. Figure 3 shows that the surface roughness increases with potential as soon as OH starts to adsorb. The surface roughness increases further upon the formation of clusters and reaches its maximum value at 1.37 V_RHE_, the potential where we observe the oxide growth front. This peak maximum is not solely due to the fact that the terrace in Figure 2E consists of two different planes, as we quantify enhanced roughness in both the higher (blue asterisk in Figure 2E and Figure 3) and lower (red asterisk in Figure 2E and Figure 3) regions, independently.

**Figure 3:**
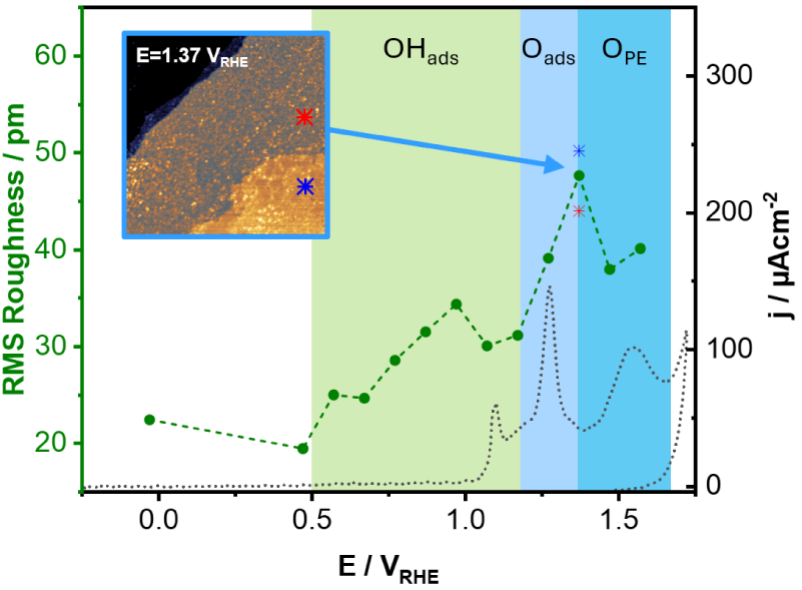
Root mean square roughness (RMS) as a function of potential. The surface roughness, calculated from the EC-STM images shown in Figure 2 and Figure S2, generally increases with potential, except for two local minima observed at 1.07 V_RHE_ and at 1.47 V_RHE_. From the image at 1.37 V_RHE_ (inset), we calculated the roughness of both the fully oxidized (darker) layer (indicated with a red asterisk) and the unoxidized (lighter) layer (indicated with a blue asterisk). Qualitatively similar trends were observed in multiple measurements despite differences in absolute values (see Figure S7).

Interestingly, the roughness decreases once the surface oxide covers the whole terrace (see Figure 3). This trend is consistent across four different sets of experiments (see Figure S7) and aligns well with previous observations in 0.1 M HClO_4_ by Gao et al. and Honbo et al., who reported an increase in roughness with potential until 1.55/1.65 V_RHE_, followed by a subsequent roughness attenuation at higher potentials.^28,29^ This unexpected decrease can be attributed to the relaxation of the oxidized surface during the irreversible place-exchange, when Au atoms are expelled onto the terraces or dissolved into the electrolyte. We discuss this in more detail in the last section.

### Reversible and Irreversible Place-Exchange

The formation of adatoms through the irreversible place-exchange as well as the Au dissolution contribute to the formation of adatom and vacancy islands and, therefore, to irreversible structural changes of the surface. The presence of adatom and vacancy islands is more evident upon reducing the oxide, as then only metallic Au is on the surface and one can assume that the differences in contrast in the EC-STM images are solely due to topography. Knowing this, we conducted an experiment to determine the onset potential for irreversible place-exchange by progressively increasing the anodic potential before reducing the surface oxide through a potential step back to 0.49 V_RHE_ (see Figure 4).

**Figure 4:**
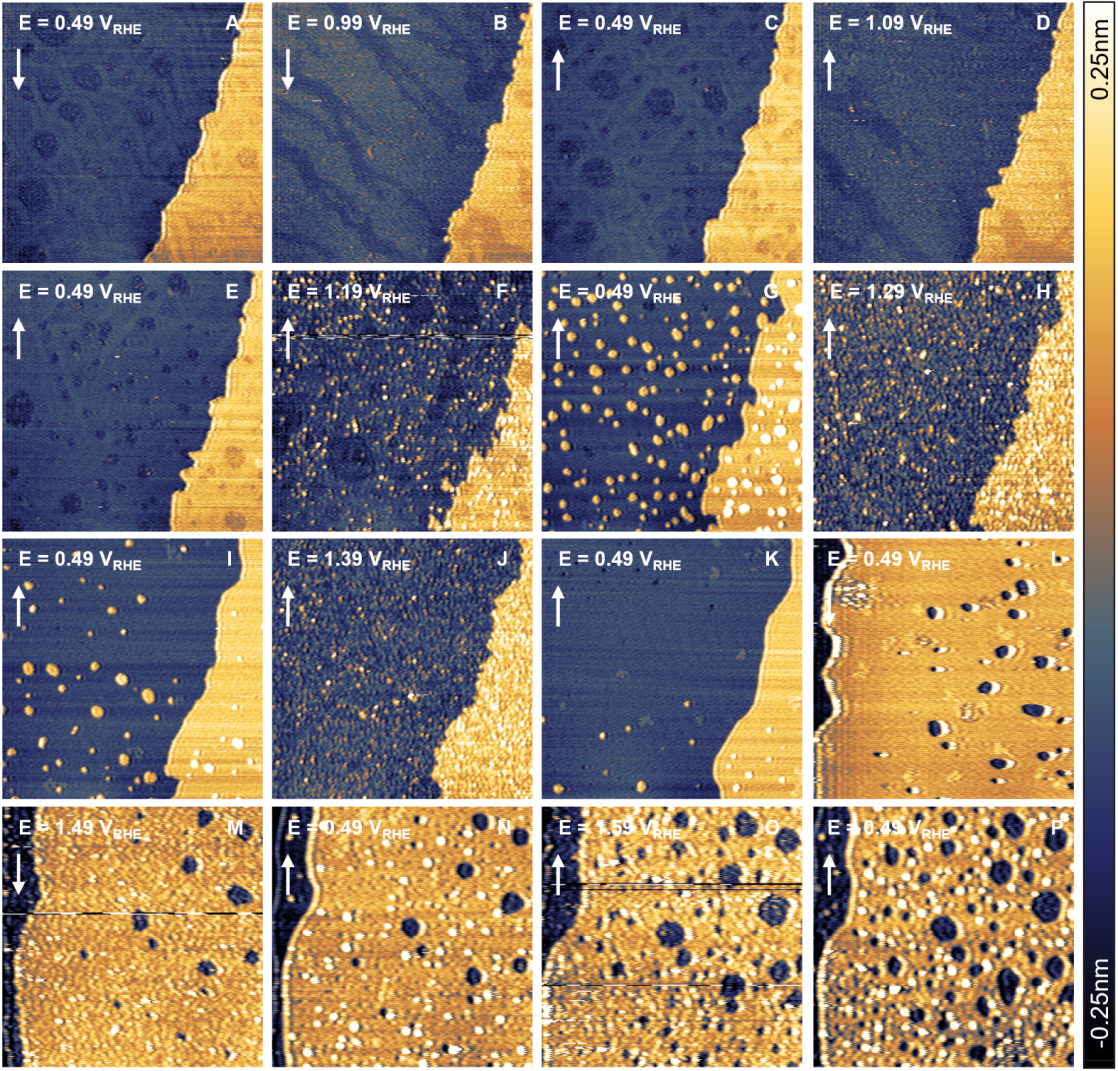
Surface (ir)­reversibility upon sequential oxidation and reduction. The anodic potential was increased progressively, while the reduction potential was always 0.49 V_RHE_. Adatom islands in (F–I) form upon the sudden lifting of the HB reconstruction, whereas adatom and vacancy islands in (M–P) result from Au atoms expelled onto the terraces during surface oxidation. For details, see the main text. From this series of measurements, we conclude that the onset of the irreversible place-exchange must be between 1.29 V_RHE_ and 1.39 V_RHE_. White arrows in the top left corner of each image indicate the slow scan direction. Each oxidation potential was applied for 2.5 min. All images are 250 × 250 nm^2^, recorded with *I*_tip_ = 1.6 nA and *E*_tip_ = 0.37 V_RHE_.

Up to 1.09 V_RHE_, anodic polarization only causes the disordering and partial lifting of the HB reconstruction, which is restored once the potential is decreased back to 0.49 V_RHE_. The complete lifting of the HB reconstruction occurs at 1.19 V_RHE_ (Figure 4F) and leads to the formation of adatoms and small adatom islands. Note that, due to tip convolution effects, it is impossible to discern between individual adatoms and small islands. Importantly, at this point the terrace oxidation has not yet begun, as this potential lies before the O_ads_ peak centered at 1.27 V_RHE_ (see CV in Figure 1B). This strongly suggests that these adatoms are not formed due to irreversible place-exchange. Upon reduction, the islands grow via ripening, which is enhanced once the oxide is fully reduced and no longer limits surface mobility (Figure 4G).

Increasing the anodic potential even further to 1.29 V_RHE_ leads to the formation of a large number of adatoms and/or small adatom islands (see Figure 4H). This high concentration suggests that the equilibrium adatom pressure significantly increases at higher potentials, when O is adsorbed on the terraces, as previously reported for Pt(111).^9^ Thus, Au atoms located at the step edges (particularly at kink sites) of the adatom islands previously formed upon reduction (Figure 4G) are expelled onto the terrace, resulting in the higher density of adatom species observed in Figure 4H. Smoothening of the step that separates the two main terraces is also observed, which indicates Au atom removal from there, as well. At the same time, Au also dissolves into the electrolyte, which explains why, once reduced, the terrace in Figure 4I shows a significantly lower adatom island density than in Figure 4G.

Figure 4J shows the Au(111) surface at 1.39 V_RHE_, where oxide clusters form with a lower density than before (Figure 4H). Upon reduction, only a few of the clusters remain, while some vacancy islands form (see Figure 4K,L). These vacancy islands result from the coalescence of individual vacancies that form as a result of the irreversible place-exchange process, in which surface atoms are expelled from the terrace and subsequently partly dissolve in the electrolyte. This vacancy formation indicates that, in 0.1 M NaOH, irreversible structural changes begin between 1.29 V_RHE_ and 1.39 V_RHE_. Therefore, the observed irreversible processes are closely associated with the oxide growth front observed at 1.37 V_RHE_ (Figure 2D). Below 1.29 V_RHE_, any place-exchange that occurs remains reversible, involving only the lifting of Au surface atoms above the surface plane without any lateral displacement.

The absence of adatom islands on the reduced surface shown in Figure 4K is a strong indication of Au dissolution, a process that starts at 1.39 V_RHE_ in 0.05 M NaOH,^39^ as well as of adatom incorporation into step edges. However, Figures 4M and 5O show the presence of numerous adatom islands at more positive potentials, such as 1.49 and 1.59 V_RHE_. Moreover, if the reduction of the surface oxide is performed rapidly, some of the dissolved Au atoms that have not diffused far into the electrolyte can redeposit onto the surface.^63^ The higher density of larger adatom islands in Figure 4N,P compared to the oxidized surface in Figure 4M,O results from two effects: dissolved Au that redeposits during reduction and adatoms that, after being immobilized by the oxide at high potentials, merge once the oxide is removed.

**Figure 5:**
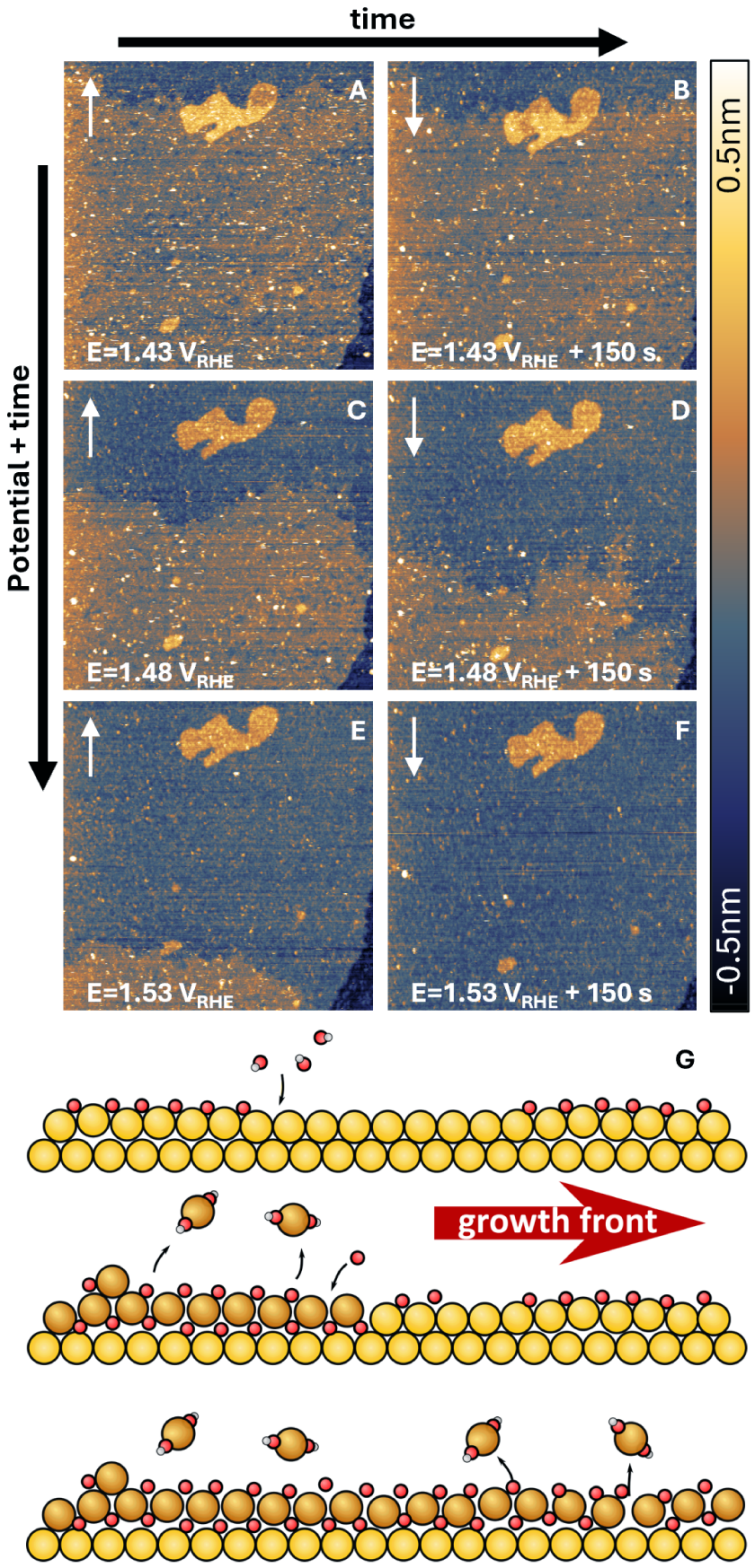
Growth of the surface oxide with potential and time. (A–F) show that, as the potential and/or oxidation time increase, the darker area advances across the terrace toward the bottom side of the image. White arrows in the top left corner of each image indicate the slow scan direction. All images are 250 × 250 nm^2^, recorded with *I*_tip_ = 1.0 nA and *E*_tip_ = 0.30 V_RHE_. (G) illustrates the suggested oxidation mechanism, which includes: O adsorption (top), the place-exchange between O_ads_ and Au surface atoms (middle), and the lateral growth of the surface oxide (middle to bottom). During this process, surface atoms are either lifted up, expelled onto the terrace, or dissolved into the electrolyte.

### Growth of the Surface Oxide on Au(111)

After identifying the onset potential for irreversible surface oxidation on Au(111), we return to the question of how this process proceeds. We could observe that surface oxidation at 1.37 V_RHE_, i.e., at potentials more positive than the first oxidation peak, leads to the formation of an area with lower apparent height in the EC-STM images that likely starts from the step edges and spreads across the terraces.^64^ To understand the dynamics and mechanism of this surface oxide growth, we examined its temporal evolution at constant potential. Figure 5 shows a series of EC-STM images displaying the growth process of the surface oxide. At 1.43 V_RHE_, the oxide layer is already formed (Figure 5A). Notably, the growth front crosses an existing adatom island, which demonstrates that this process is not confined to pristine terrace regions but proceeds regardless of preexisting surface features. The dark region continues to expand over time, even when the potential is held constant (Figure 5A–B, Figure 5C–D, and Figure 5E–F). This demonstrates that the observed oxidation process is kinetically limited.^30,52^

Figure 5G provides a plausible model for the oxidation mechanism based on our observations. Above 1.2 V_RHE_, O adsorption begins, as indicated by the corresponding anodic peak in Figure 1. This leads to the formation of a surface dipole, where the negative charge resides on the O_ads_, while the Au atoms carry a positive sign. The combination of this surface dipole and the high electric field drives the place-exchange process, in which O_ads_ moves subsurface while Au surface atoms are displaced upward.^4,8,52,65^ Most likely, this is the slow step that kinetically hinders the oxidation of the surface.

The formation of the surface oxide results in a reduction of the LDOS, leading to a darker contrast in STM images compared to the pristine Au surface. As this oxide grows, the surface stress increases due to the lattice mismatch between the bare gold and the oxide formed. In order to decrease this stress, the surface relaxes by expelling Au surface atoms onto the terrace via the irreversible place-exchange process,^9,23,24,55^ which leads to the formation of clusters.^26,28,29,31^ In addition, some Au dissolves into the electrolyte, likely as [Au­(OH)_2_]^−^. While the formation of adatoms increases surface roughness, both Au dissolution and the relaxation of partially lifted surface atoms back into the terrace plane counterbalance this effect. This interplay results in the overall reduction of the roughness observed at 1.47 V_RHE_, as shown in Figure 3.

A notable feature of the observed surface oxide is that it grows as a single, large domain rather than from multiple small nuclei distributed across the terrace. This growth mode likely reflects distinct factors that govern nucleation versus propagation. Although entropy would favor the formation of many small nuclei, preferential nucleation at defect sites, and the migration of these defect sites across the surface as place-exchange proceeds,^5^ can facilitate oxide growth on already existing nuclei. Additionally, once a critical density of surface dipoles develops, their aggregation into larger domains may be energetically more favorable than distributing individual dipoles across the surface.^16,66^ Once nucleated, several factors favor growth at existing oxide/metal boundaries rather than new nucleation. Atoms lifted during oxidation leave neighboring atoms undercoordinated, lowering the activation barrier for continued place-exchange at the boundary. Furthermore, strain from lattice mismatch between oxide and metallic Au is more efficiently accommodated by extending existing interfaces rather than by creating new high-energy boundaries elsewhere.

We cannot completely rule out the possibility of smaller oxide patch formation on larger terraces that have not been directly imaged in this study. Nonetheless, the occurrence of the oxide growth front has been observed four times throughout this study and is therefore certainly meaningful and significant. The formation mechanism of the surface oxide and its initial growth still remains the subject of ongoing investigation and will be addressed in future work.

## Conclusions

In this work, we used EC-STM to investigate the electrochemical oxidation of Au(111) in 0.1 M NaOH through analysis of the structural and morphological evolution of the Au(111) surface with increasing anodic potential.

At sufficiently high potentials (≥1.37 V_RHE_), surface oxidation is characterized by the formation of a Au oxide layer that propagates as a distinct front across the terraces. This oxide layer appears as a darker, less conductive region in EC-STM images, reflecting changes in the electronic structure due to oxygen incorporation. The slow growth of the surface oxide under constant potential suggests that this process is kinetically limited, driven by structural rearrangements (i.e., the place-exchange between O and the underlying Au surface atoms) rather than O adsorption alone. The lattice mismatch between the surface oxide formed and the Au substrate beneath induces surface stress, which is released by expelling Au atoms onto the terrace via the so-called irreversible place-exchange or into the electrolyte (dissolution).

Reducing the oxide formed at (or above) 1.39 V_RHE_ results in adatom and vacancy island formation, and is thus irreversibly altering the pristine Au(111) surface. By contrast, maintaining the potential below 1.09 V_RHE_ enables full structural restoration of the surface upon reduction, including reformation of the HB reconstruction, which is not fully lifted at potentials below 1.29 V_RHE_. Vacancy island formation unraveled the potential range between 1.29 and 1.39 V_RHE_ as the onset of the irreversible place-exchange process. Notably, this potential range also coincides with the onset of Au dissolution in alkaline media, as demonstrated in previous studies. It is also in this potential range that the oxide growth front is observed.

Overall, our findings advance the understanding of the Au(111) oxidation process dynamics in alkaline electrolytes and clarify the interplay between oxide growth, place-exchange, and dissolution. These insights are directly relevant to electrocatalytic processes such as the direct CO and alcohol oxidation, where the stability of the Au surface governs long-term activity. The understanding of the surface structure evolution during changes of the electrode potential provides a foundation for developing strategies to mitigate roughening and extend the operational lifetime of Au-based electrocatalysts.

## Supplementary Material



## Data Availability

The data that support the findings of this study are openly available in InvenioRDM at 10.48323/rwakc-a2940

## Notes

Additional EC-STM images of the oxidation and reduction; further details on the roughness analysis (PDF)
