# Supplementary material for: Oxide Growth and Place-Exchange on Au(111) in Alkaline Electrolyte
Source: ACS Electrochem. 2026 Feb 3;2(3):726–34. doi: 10.1021/acselectrochem.5c00505 (PMC12969257; doi:10.1021/acselectrochem.5c00505)
Supplement: Supplementary file 1 [file ec5c00505_si_001.pdf]

Supporting Information

Oxide Growth and Place-Exchange on Au(111) in Alkaline Electrolyte

Toni Moser[1], Francesc Valls Mascaró[1], Andrea Auer[1], Julia Kunze-Liebhäuser[1*]

[1]University of Innsbruck, Innrain 52c, 6020 Innsbruck, Austria

(*corresponding author: Julia Kunze-Liebhäuser, email: julia.kunze@uibk.ac.at)

Table of Contents

Figure S1. Cyclic voltammograms (CVs) and oxidation charges of Au(111) in acidic and alkaline media. 1

Figure S2. Height lines over the step edge and the OH_{ads} patches. 2

Figure S3. Structural evolution of the Au(111) surface upon anodic polarization. 3

Figure S5. Formation of adatom islands upon abrupt lifting of the Herringbone reconstruction. 6

Figure S6. Height profiles over oxide clusters and the oxidation front at 1.27 V_{RHE} and 1.37 V_{RHE}. 7

Supporting Note 1. Quantification of Root mean square roughness. 7

Figure S7. Root mean square roughness as function of oxidation potential. 8

References 8

Supporting Figures

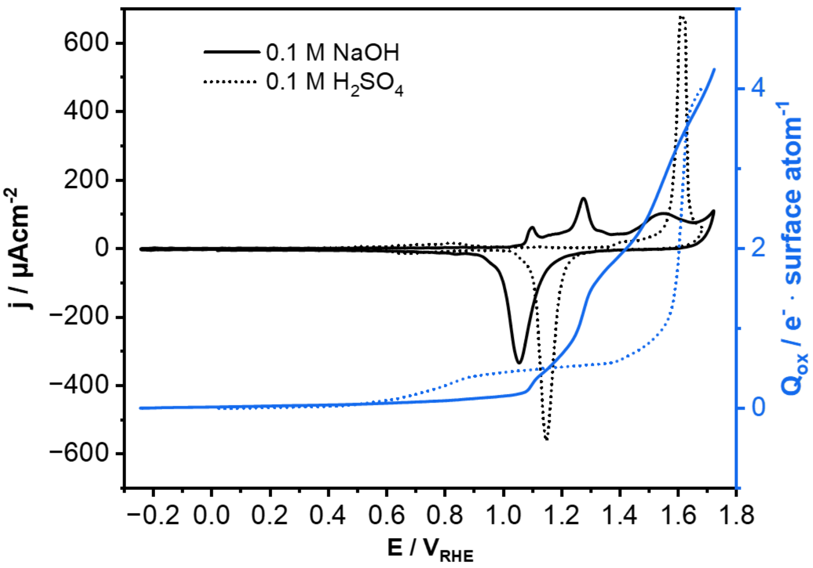

Figure S1. Cyclic voltammograms (CVs) and oxidation charges of Au(111) in acidic and alkaline media. The solid black curve represents the CV in 0.1 M NaOH, while the dotted black curve the CV in 0.1 M H_2SO_4. The corresponding oxidation charges, shown in blue, were extracted from the CVs and normalized to the 1×1 unreconstructed surface. Interestingly, although the oxidation fingerprint is significantly different in acidic and alkaline media, the total oxidation charge reaches similar values at 1.7 V_{RHE}. This is consistent with the notion that, at a given potential vs. RHE, the equilibrium surface coverage of oxidized species should be similar regardless of the reaction pathway or pH.

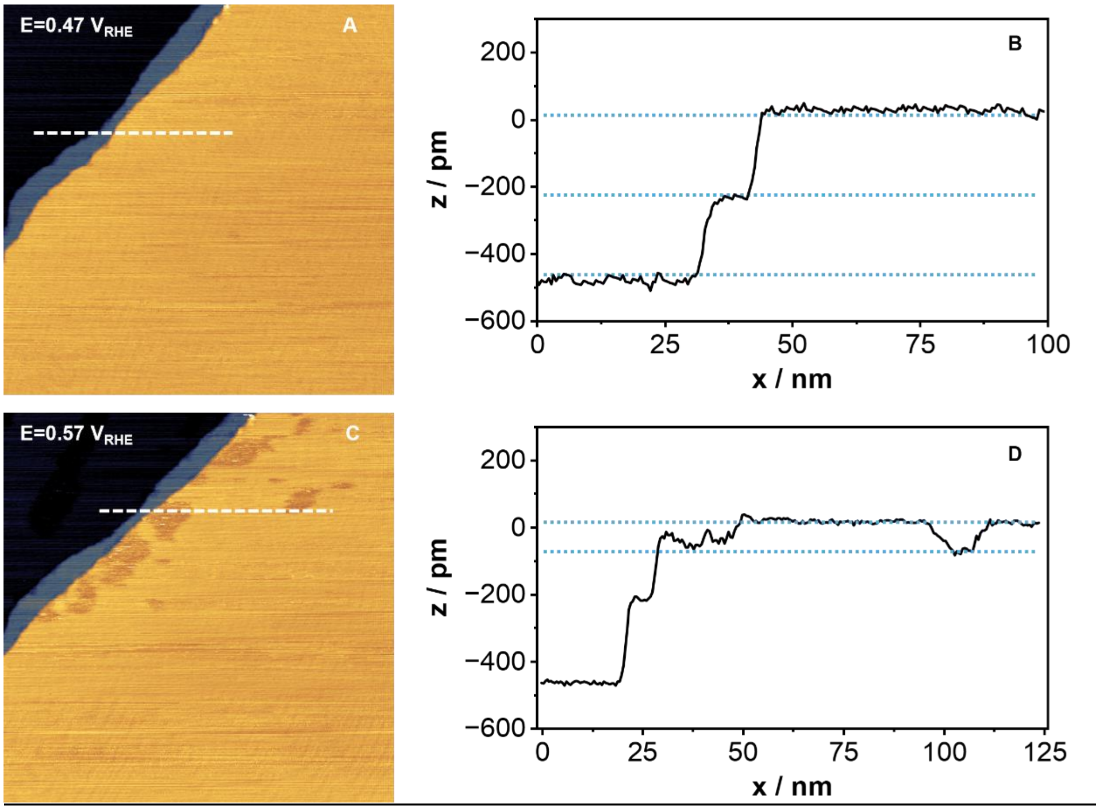

Figure S2. Height lines over the step edge and the OH_{ads} patches. The step height is approximately 0.24 nm, matching the theoretical value, while the apparent depth of the darker patches is approximately 0.08 nm.

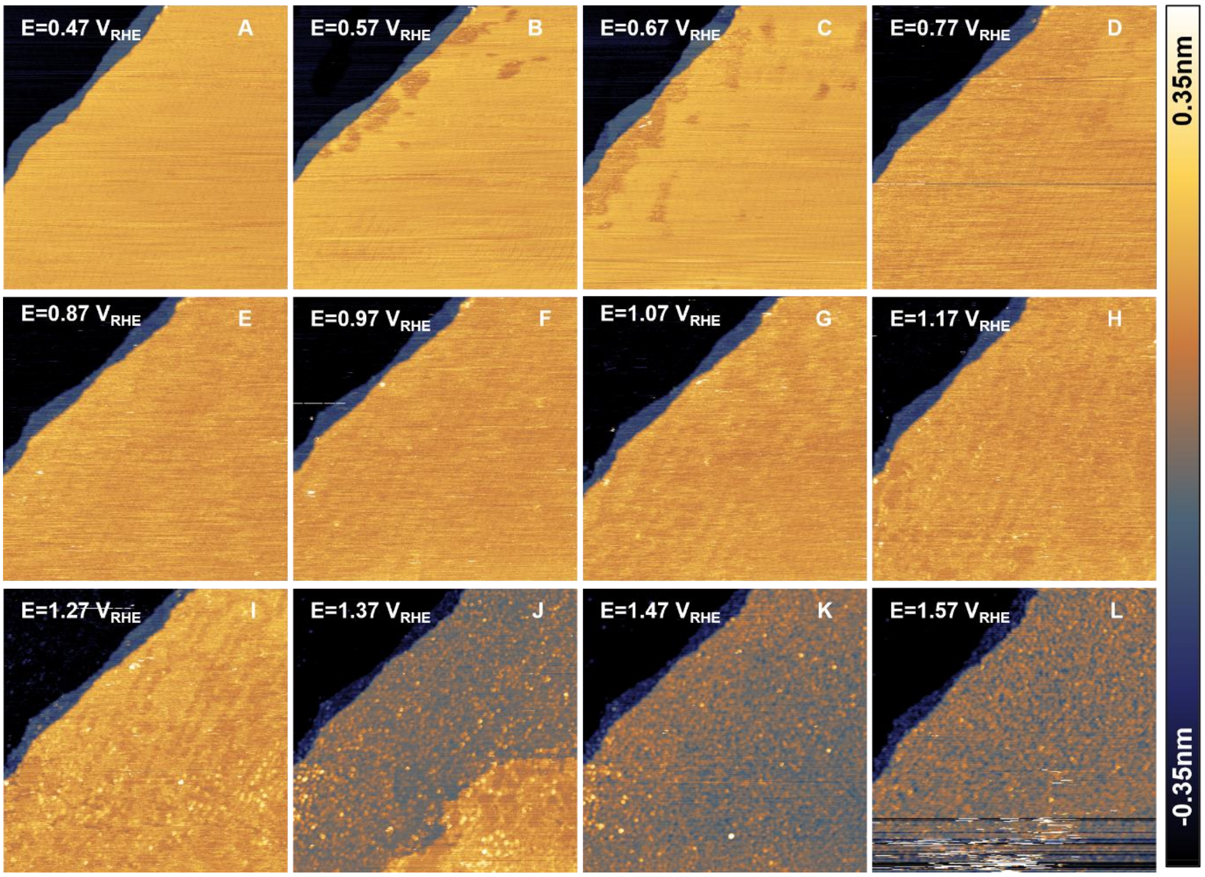

Figure S3. Structural evolution of the Au(111) surface upon anodic polarization. EC-STM images recorded in 0.1 M NaOH at (A) 0.47 V_{RHE}, (B) 0.57 V_{RHE}, (C) 0.67 V_{RHE}, (D) 0.77 V_{RHE}, (E) 0.87 V_{RHE}, (F) 0.97 V_{RHE}, (G) 1.07 V_{RHE}, (H) 1.17 V_{RHE}, (i) 1.27 V_{RHE}, (J) 1.37 V_{RHE}, (K) 1.47 V_{RHE}, (L) 1.57 V_{RHE}. All images are 200 × 200 nm², recorded with I_{tip} = 1.8 nA and E_{tip} = 0.47 V_{RHE}.

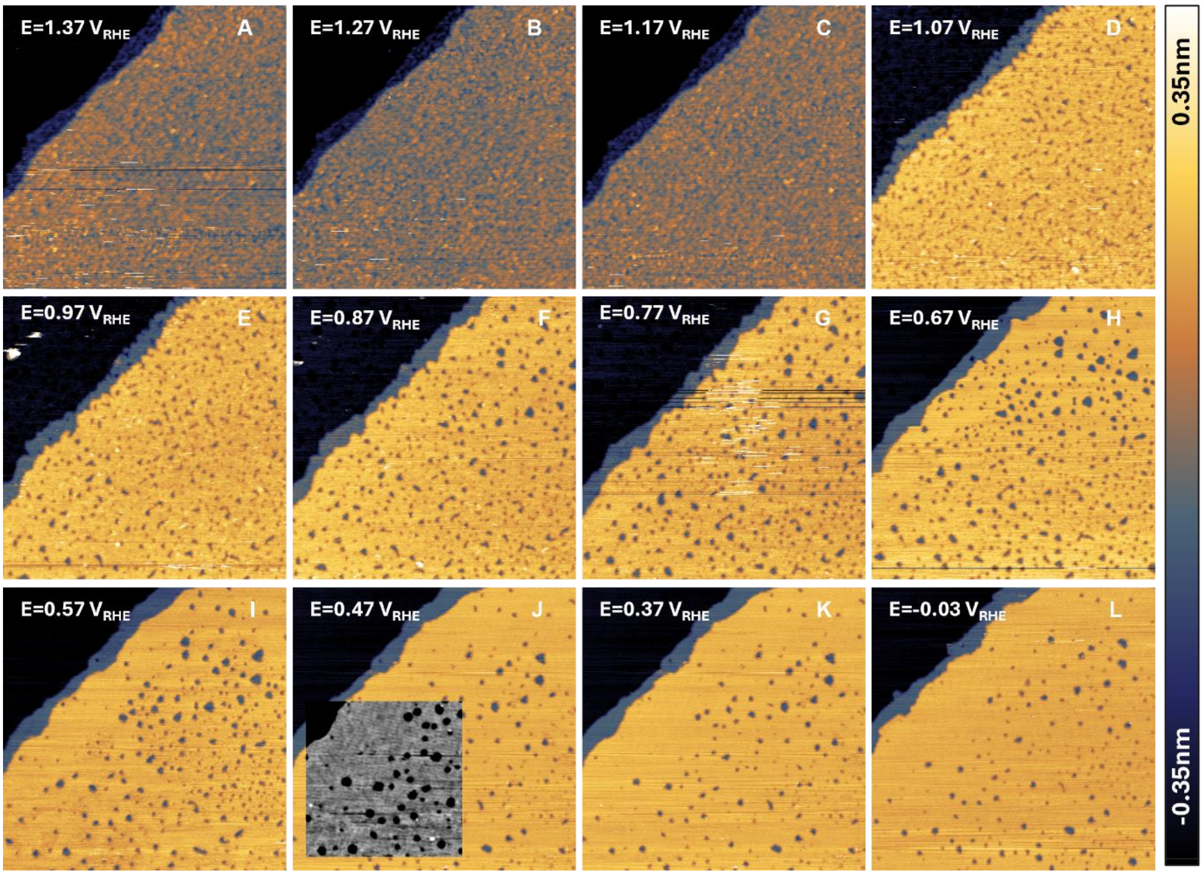

Figure S4. Structural evolution of the Au(111) surface upon reduction of the oxide. EC-STM images recorded in 0.1 M NaOH at (A) 1.37 V_{RHE}, (B) 1.27 V_{RHE}, (C) 1.17 V_{RHE}, (D) 1.07 V_{RHE}, (E) 0.97 V_{RHE}, (F) 0.87 V_{RHE}, (G) 0.77 V_{RHE}, (H) 0.67 V_{RHE}, (I) 0.57 V_{RHE}, (J) 0.47 V_{RHE}, (K) 0.37 V_{RHE}, (L) -0.03 V_{RHE}. The grayscale inset in (J) is an overlay of the same area with increased contrast. All images are 200 × 200 nm², recorded with I_{tip} = 1.8 nA and E_{tip} = 0.47 V_{RHE}.

Figure S4 shows that the reduction of the surface oxide occurs below 1.17 V_{RHE}, which is in full agreement with the cathodic peak in the CV (Fig. S1). At 1.07 V_{RHE} (Fig. S4 D), the surface exhibits flat terraces with monoatomically deep vacancy islands of irregular shapes, which indicates significant oxide reduction. During the reduction process, lifted Au atoms relax back into the surface, while Au adatoms and adatom islands (previously Au oxide) lose their anchoring to the surface due to the removal of O_{ads}, which enhances their mobility[1]. These

adatoms may either nucleate into Au adatom islands or become incorporated into step edges. Additionally, some of them dissolve into the electrolyte, as confirmed by ICP-MS measurements[2–4]. All three processes, adatom island formation, incorporation into step edges, and dissolution, lead to the formation of vacancies on the surface, which can coalesce into vacancy islands.

However, the absence of adatom islands in Fig. S4 D-L confirms that, under the measurement conditions used, all adatoms either dissolve into the electrolyte or migrate to step edges. This observation contrasts with many EC-STM studies conducted in acidic media, where adatom islands are commonly observed during reduction[5–8] . This discrepancy can be attributed to two key factors. First, surface mobility is significantly enhanced in alkaline media, as we demonstrated in a recent publication where the surface evolution during oxidation and reduction of Au(111) in different electrolytes is compared.[9] Secondly, as shown by Stumm et al., a slow reduction of the surface oxide promotes Au dissolution into the electrolyte, further decreasing the density of adatom islands.[4] Actually, Fig. S8 shows that, even in alkaline media, adatom islands can form if the potential is reduced rapidly from 1.49 V_{RHE} to 0.49 V_{RHE}. This emphasizes the critical influence of kinetics on the resulting morphology of the Au(111) surface.

Typically, vacancies are less mobile than adatoms, which explains the high density of vacancy islands observed in Fig. S4 D[10]. Decreasing the potential to 0.47 V_{RHE} causes the complete reduction of the surface oxide, leading to the formation of new vacancies as well as the growth of preexisting ones. The latter is favored by Ostwald and Smoluchowski ripening[11,12]. These ripening processes are evident in Fig. S4 D-L, where the vacancy island density diminishes significantly while their size increases. Additionally, vacancy islands in the region near the descending step edge disappear, which evidences that vacancies formed nearby have been incorporated into the step edge[13]. Interestingly, in this flat region the HB reconstruction

reappears, as shown in the inset of Fig. S4 J, which provides an image with enhanced contrast[14–16].

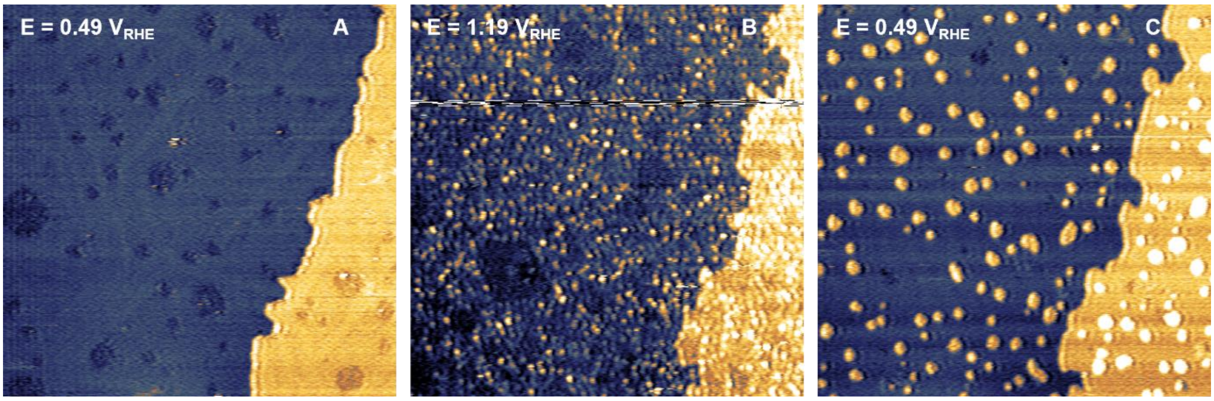

Figure S5. Formation of adatom islands upon abrupt lifting of the Herringbone reconstruction. Potential jump from (A) 0.49 V_{RHE} to (B) 1.19 V_{RHE} and back to (C) 0.49 V_{RHE}, as indicated. All images are 250 × 250 nm², recorded with I_{tip} = 1.6 nA and E_{tip} = 0.37 V_{RHE}.

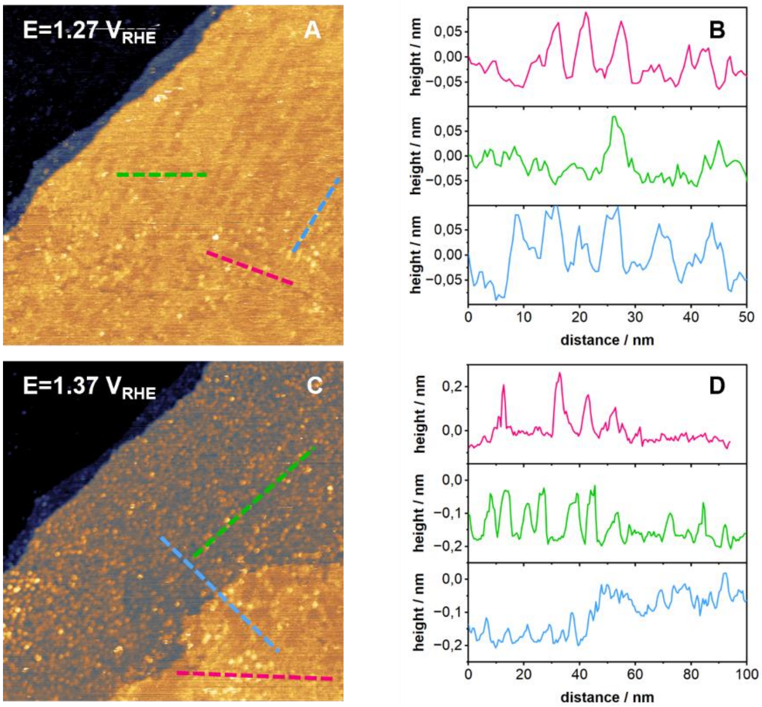

Figure S6. Height profiles over oxide clusters and the oxidation front at 1.27 VRHE and 1.37 VRHE. The dashed lines correspond to the height lines shown on the right side.

Supporting Note 1. Quantification of Root mean square roughness.

The EC-STM images were processed by applying a plane correction over the largest terrace. After this, the root mean square roughness on this terrace was quantified by:

$$RMS\ Roughness = \sqrt{\frac{\sum_i (h_i - \bar{h_i})^2}{n}}$$

Where h_i is the height at pixel i, $\bar{h_i}$ is the mean height over all the pixels at the terrace, and n is the total number of pixels.

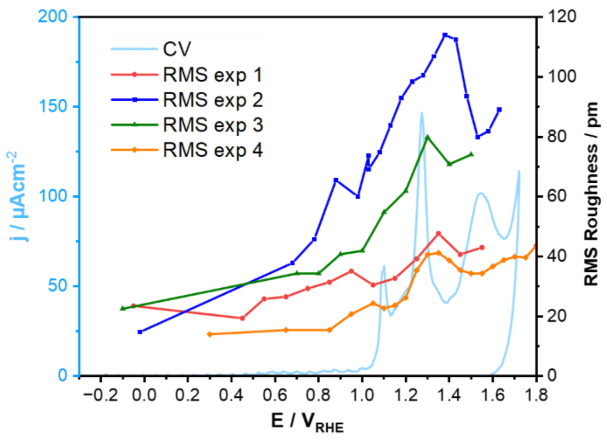

Figure S7. Root mean square roughness as function of oxidation potential. Data from four independent measurements show a local roughness maximum near 1.35 V_{RHE}.

References

(1) Valls Mascaró, F.; McCrum, I. T.; Koper, M. T. M.; Rost, M. J. Nucleation and Growth of Dendritic Islands during Platinum Oxidation-Reduction Cycling. *J. Electrochem. Soc.* **2022**, *169* (11). DOI: 10.1149/1945-7111/ac9bdb.

(2) Cherevko, S.; Topalov, A. A.; Zeradjanin, A. R.; Katsounaros, I.; Mayrhofer, K. J. J. Gold dissolution: towards understanding of noble metal corrosion. *RSC Adv.* **2013**, *3* (37). DOI: 10.1039/c3ra42684j.

(3) Cherevko, S.; Zeradjanin, A. R.; Keeley, G. P.; Mayrhofer, K. J. J. A Comparative Study on Gold and Platinum Dissolution in Acidic and Alkaline Media. *J. Electrochem. Soc.* **2014**, *161* (12). DOI: 10.1149/2.0881412jes.

(4) Stumm, C.; Grau, S.; Speck, F. D.; Hilpert, F.; Briega-Martos, V.; Mayrhofer, K.; Cherevko, S.; Brummel, O.; Libuda, J. Reduction of Oxide Layers on Au(111): The Interplay between Reduction Rate, Dissolution, and Restructuring. *J. Phys. Chem. C* **2021**, *125* (41). DOI: 10.1021/acs.jpcc.1c03969.

(5) Gao, X.; Weaver, M. J. Nanoscale structural changes upon electro-oxidation of Au(111) as probed by potentiodynamic scanning tunneling microscopy. *Journal of Electroanalytical Chemistry* **1994**, *367* (1-2). DOI: 10.1016/0022-0728(93)03263-O.

(6) Hanson, K. J.; Green, M. P. Electrochemical Roughening and Annealing of Au(111) Surfaces in Perchloric and Sulfuric Acid Electrolytes Studied by STM. *MRS Proc.* **1991**, *237.* DOI: 10.1557/PROC-237-323.

(7) Rodríguez Nieto, F. J.; Andreasen, G.; Martins, M. E.; Castez, F.; Salvarezza, R. C.; Arvia, A. J. Scanning Tunneling Microscopy, Voltammetry, and X-ray Photoelectron Spectroscopy Study of the Early Stages of Electrochemical Faceting of Gold (111) in Aqueous Sulfuric and Perchloric Acid. *J. Phys. Chem. B* **2003**, *107* (41). DOI: 10.1021/jp0353542.

(8) Schneeweiss, M. Oxide formation on Au(111) an in situ STM study:An in situ STM study. *Solid State Ionics* **1997**, *94* (1-4). DOI: 10.1016/S0167-2738(96)00587-5.

(9) Moser, T.; Valls Mascaró, F.; Kunze-Liebhäuser, J. Dynamics of Adatom and Vacancy Islands on Au(111) in Alkaline and Acidic Media. *J. Phys. Chem. C* **2025**, *129* (32). DOI: 10.1021/acs.jpcc.5c03661.

(10) Stoltze, P. Simulation of surface defects. *J. Phys.: Condens. Matter* **1994**, *6* (45). DOI: 10.1088/0953-8984/6/45/004.

(11) Morgenstern, K.; Rosenfeld, G.; Lægsgaard, E.; Besenbacher, F.; Comsa, G. Measurement of Energies Controlling Ripening and Annealing on Metal Surfaces. *Phys. Rev. Lett.* **1998**, *80* (3). DOI: 10.1103/PhysRevLett.80.556.

(12) Rosenfeld, G.; Morgenstern, K.; Esser, M.; Comsa, G. Dynamics and stability of nanostructures on metal surfaces. *Applied Physics A: Materials Science & Processing* **1999**, *69* (5). DOI: 10.1007/s003390051448.

(13) Rost, M. J.; Jacobse, L.; Koper, M. T. M. The dualism between adatom- and vacancy-based single crystal growth models. *Nature communications* **2019**, *10* (1). DOI: 10.1038/s41467-019-13188-0.

(14) Kondo, T.; Morita, J.; Hanaoka, K.; Takakusagi, S.; Tamura, K.; Takahasi, M.; Mizuki, J.; Uosaki, K. Structure of Au(111) and Au(100) Single-Crystal Electrode Surfaces at Various Potentials in Sulfuric Acid Solution Determined by In Situ Surface X-ray Scattering. *J. Phys. Chem. C* **2007**, *111* (35). DOI: 10.1021/jp072601j.

(15) Kondo, T.; Zegenhagen, J.; Takakusagi, S.; Uosaki, K. In situ real-time study on potential induced structure change at Au(111) and Au(100) single crystal electrode/sulfuric acid solution interfaces by surface x-ray scattering. *Surface Science* **2015**, *631.* DOI: 10.1016/j.susc.2014.06.013.

(16) Adnan, A.; Behjati, S.; Félez-Guerrero, N.; Ojha, K.; Koper, M. T. M. Tracking the surface structure and the influence of cations and anions on the double-layer region of a Au(111) electrode. *Physical chemistry chemical physics : PCCP* **2024**, *26* (32). DOI: 10.1039/d4cp02133a.
